# Predictors of maternal and neonatal outcomes in labors complicated by shoulder dystocia: a comparative analysis

**DOI:** 10.1007/s00404-024-07663-3

**Published:** 2024-08-06

**Authors:** Daniel Tairy, Shalhevet Frank, Shir Lev, Yael Ganor Paz, Jacob Bar, Giulia Barda, Eran Weiner, Michal Levy

**Affiliations:** Departments of Obstetrics and Gynecology, The Edith Wolfson Medical Center Holon, Israel Affiliated with Sackler Faculty of Medicine, Tel Aviv University, P.O. Box 5, Holon 58100, Tel Aviv, Israel

**Keywords:** Shoulder dystocia, Predictors, Maternal outcomes, Neonatal outcomes

## Abstract

**Introduction:**

Studies investigating the risk factors associated with unfavorable maternal/neonatal outcomes in cases of shoulder dystocia are scarce. This study aims to uncover the predictive factors that give rise to unfavorable outcomes within the context of shoulder dystocia.

**Materials and methods:**

Medical records of pregnancies complicated by shoulder dystocia was obtained between 2008–2022 from a single tertiary center. This study involved the comparison of sociodemographic, sonographic, and delivery characteristics among pregnancies complicated by shoulder dystocia resulting in favorable vs. unfavorable maternal/neonatal outcomes.

**Results:**

A total of 275 pregnancies were analyzed, with 111 (40.3%) classified as unfavorable outcomes and 164 (59.7%) as favorable outcomes. Employing a multivariable regression analysis, several independent associations were identified with unfavorable maternal/neonatal outcomes. Specifically, short maternal stature, pre-gestational diabetes, vacuum extraction, Wood’s screw maneuver, and macrosomia merged as significant predictors of unfavorable maternal/neonatal outcomes.

**Conclusion:**

Short maternal stature, pre-gestational diabetes, vacuum extraction, Wood’s screw maneuver, and macrosomia may all contribute to poor maternal/neonatal outcomes in shoulder dystocia cases. This knowledge allows clinicians to improve their decision-making, patient care, and counseling.

## What does this study add to the clinical work

**Table:** 

Maternal shorter stature, pregestational diabetes, vacuum extraction, Wood's screw maneuver, and neonatal macrosomia are contributing factors to adverse maternal/neonatal outcomes in instances of shoulder dystocia.	

## Introduction

Shoulder dystocia is a term used to describe a vaginal delivery in which after delivery of the fetal head, additional obstetric maneuvers are needed to enable delivery of the fetal shoulders [1].

This condition occurs in 0.2–3% of births [2] and presents a range of risk factors, which can be categorized as either antepartum or intrapartum. Antepartum risks encompass elements like high birth weight, maternal obesity, excessive gestational weight gain, diabetes, advanced maternal age at first birth, previous shoulder dystocia, post-term pregnancies, and male fetal sex [3–9]. Intrapartum risk factors include factors like abnormal labor progress and interventions such as assisted vaginal delivery [10–13].

The implications of shoulder dystocia extend to neonatal complications, including brachial plexus palsy, clavicular/humerus fractures, hypoxic–ischemic encephalopathy, and even intrapartum fetal death and neonatal mortality [14–17]. In terms of maternal consequences, the impact is also marked by significant morbidities, prominently characterized by instances of hemorrhage and perineal lacerations ranging from third to fourth degree [18, 19].

Studies investigating the risk factors associated with unfavorable maternal/neonatal outcomes in cases of shoulder dystocia are scarce. These studies specifically investigated risk factors associated with shoulder dystocia cases, focusing on specific neonatal morbidities like permanent brachial plexus palsy [20, 21]. Given the limited research on diverse adverse maternal/neonatal outcomes related to shoulder dystocia, there exists a significant knowledge gap. To bridge this gap, our study aims to offer valuable insights to clinicians by investigating the contributing factors to these unfavorable outcomes. By pinpointing the specific characteristics and circumstances linked to adverse outcomes, clinicians can customize their approaches for individual patients and provide more comprehensive counseling to expectant parents.

## Materials and methods

### Study population

In this retrospective study, the medical records, sonographic reports, delivery charts, and neonatal charts of all singleton deliveries which resulted in shoulder dystocia between December 2008 and April 2022 from a single university hospital were reviewed. We excluded multiple pregnancies, terminations of pregnancy, cases of prior to delivery intrauterine fetal deaths, pregnancies with a known major fetal malformation, and cases with missing data. Shoulder dystocia was diagnosed in a vaginal delivery in which after delivery of the fetal head, additional obstetric maneuvers were needed to enable delivery of the fetal shoulders.

The study population was further divided into two groups allowing for a clear differentiation between cases leading to adverse outcomes and those resulting in more favorable results within the study population:

The “Unfavorable Outcome” group encompassed cases complicated by any of the following conditions: maternal—perineal tear grade 3/4, postpartum fever, postpartum hemorrhage, or blood transfusion; neonatal—Apgar score at 5 min < 7, umbilical pH < 7.1, Erb’s palsy, clavicular fracture, cerebral complications (intra-ventricular hemorrhage, seizures or hypoxic–ischemic encephalopathy), respiratory complications (respiratory distress syndrome, mechanical ventilation or need for respiratory support), necrotizing enterocolitis, sepsis, blood transfusion, or neonatal death.

The “Favorable Outcome” group comprised all other cases that did not meet the criteria for unfavorable outcomes.

Maternal demographics, sonographic parameters, and delivery characteristics were compared between deliveries complicated with shoulder dystocia, which resulted with either an unfavorable or favorable outcomes.

## Ethical approval and informed consent

All procedures were in accordance with the ethical standards of the institutional Review Board and with the 1964 Helsinki declaration and its later amendments or comparable ethical standards. Decision number 0013–22-WOMCE dated 18/01/2022. Since the study used anonymous archival data and was retrospective in nature, informed consent was not obtained.

## Data collection

The following maternal characteristics were collected from the medical chart of the patients: maternal age, maternal stature, pre-pregnancy weight and height (from which the body mass index (BMI) was calculated), diabetes mellitus, hypertensive disorder, smoking, nulliparity, and previous shoulder dystocia. Short maternal stature was defined as height less than 160 cm. Although a height of less than 155 cm is commonly accepted as the threshold for short stature, only 5.2% of the women in our study were below this height. To ensure a sufficient sample size for meaningful analysis, we set the cutoff value for short stature at less than 160 cm.

Sonographic characteristics were collected as well and included: fetal weight estimation, abdominal circumference > 90%, head circumference/ abdomen circumference ratio.

Delivery characteristics were collected as well and included: gestational age at delivery, second stage of labor > 1 h, vacuum extraction, the use of maneuvers including McRoberts, suprapubic pressure, posterior shoulder extraction, Rubin and wood’s screw maneuver, shoulder dystocia time ≥ 90 s (in cases in which the time from head to shoulders delivery was documented in the medical record), induction of labor, episiotomy, epidural analgesia, neonatal weight, macrosomia, and whether the delivery took place on a night shift.

Gestational age was calculated based on the woman’s first ultrasound examination in the pregnancy and last menstrual period [22]. A woman was considered to have diabetes mellitus if she had a diagnosis of type 1/type 2 in the medical record or gestational diabetes mellitus based on the two-step approach described in the National Diabetes Group criteria [23]. Chronic hypertension and preeclampsia were diagnosed according to the current American College of Obstetricians and Gynecologists criteria [24] which were fully adapted by our institution for hypertensive disorders diagnosis and management.

Immediately after birth, all neonates were examined by pediatricians. Birth weight percentiles for gestational age were assigned using the updated local growth charts [25]. Macrosomia was defined as actual birthweight ≥ 90th percentile for gestational age/absolute weight ≥ 4000 g.

In all cases, estimated fetal weight and Doppler findings were determined at the departmental ultrasound unit < 7 days from delivery, by expert sonographers. All ultrasound examinations were performed using the Voluson series ultrasound machines (E6, E8, E10, GE Healthcare, Gretz Ultrasound, Zipf, Austria) using a 6 MHz transabdominal transducer. The following data were collected from the sonographic records: gestational age at sonographic study, abdominal circumference, head circumference, biparietal diameter, femur length from which the estimated fetal weight was calculated [26].

The following data were collected from the neonatal records: birthweight, cord blood pH, Apgar score, respiratory morbidity (defined as—respiratory distress syndrome/need for mechanical ventilation or support), cerebral morbidity (defined as—intra-ventricular hemorrhage, hypoxic ischemic encephalopathy, seizures), hypoglycemia, necrotizing enterocolitis, sepsis, need for blood transfusion, and death.

The following data were collected from the maternal post-delivery records: perineal tear grade 3/4, postpartum fever, postpartum hemorrhage (defined as estimated blood loss > 500 ml), blood transfusion.

## Data analysis

Data analysis was performed utilizing SPSS software version 22.0 (SPSS Inc., Chicago, IL, USA). Data were presented as follows: continuous variables are presented either as mean ± SD or as median and range, as appropriate. Categorical variables are presented as *n* (%). Continuous parameters were compared by the Student *t *test and categorical variables by the Chi^2^ with Yates’ correction test or the Fisher exact test, as appropriate. A statistically significant *p* < 0.05 was defined.

A multivariable regression analysis was performed in which composite of unfavorable maternal/ neonatal outcome served as the dependent variable while short maternal stature, BMI, pre-gestational diabetes, smoking, vacuum extraction, wood's screw maneuver, shoulder dystocia ≥ 90 s, and macrosomia served as independent variables.

## Results

A total of 275 deliveries were included in the study: 111 (40.3%) in the unfavorable outcome group and 164 (59.6%) in the favorable outcome group. In our institution, there are approximately 3,600 vaginal deliveries per year, which corresponds to 50,4000 delivers in the study period. The rate of shoulder dystocia in our institution was approximately 0.5% (275/50400).

Maternal and sonographic characteristics of the two groups are presented and compared in Table 1. Patients in the unfavorable outcome group were shorter, had higher BMI, higher rate of pre-gestational diabetes, and smoking.

**Table 1:** Maternal and sonographic characteristics of the study groups

	Unfavorable outcome *n* = 111	Favorable outcome *n* = 164	*p* value
Maternal characteristics			
Age (years)	31.1 ± 5	31.1 ± 5.2	1
Short maternal stature (≤ 160 cm)	41/74 (55.4)	40/115 (34.8)	**< 0.01**
BMI (kg/m^2^)	25.95 ± 6.3	24.84 ± 4.5	**0.05**
Obesity (BMI > 30 kg/m^2^)	14/61 (23.0)	12/88 (13.6)	0.14
GDMA1	13 (11.7)	16 (9.8)	0.6
GDMA2	3 (2.7)	9 (5.5)	0.26
Pre-gestational diabetes	7 (6.3)	3 (1.8)	**0.05**
Hypertensive disorder	5 (4.5)	4 (2.4)	0.34
Preeclampsia	6 (5.4)	3 (1.8)	0.10
Smoking	17/110 (15.5)	11/157 (7.0)	**0.02**
Nulliparity	29 (26.1)	31 (18.9)	0.15
Previous shoulder dystocia	3 (2.7)	0 (0)	0.06
Sonographic characteristics prior to delivery			
Fetal weight estimation (g)	3418 ± 541.8	3444.9 ± 442.5	0.65
Abdomen circumference > 90%	19/48 (39.6)	20/50 (40)	0.96
Head circumference/abdomen circumference	0.94 ± 0.05	0.93 ± 0.05	0.1

All data are shown as number (%), mean ± standard deviation or median (range), as appropriate. *BMI* body mass index. Values in bold are statistically significant (*p* < 0.05)

There were no differences in sonographic parameters between the study groups.

Delivery characteristics of the two groups are presented and compared in Table 2. Patients in the unfavorable outcome group had higher rate of vacuum extraction, Wood’s screw maneuver, shoulder dystocia time ≥ 90 s, and macrosomia.

**Table 2:** Delivery characteristics of the study groups

	Unfavorable outcome *n* = 111	Favorable outcome *n* = 164	*p* value
Gestational age at delivery (weeks)	39.4 ± 1.3	39.6 ± 1.2	0.19
Second stage of labor > 1 h	20/108 (18.5)	20/158 (12.7)	0.18
Vacuum extraction	19 (17.1)	12 (7.3)	**0.01**
McRoberts maneuver	100 (90.1)	145 (87.9)	0.66
Suprapubic pressure	96 (86.5)	148 (89.7)	0.33
Posterior shoulder extraction	12 (10.8)	9 (5.5)	0.10
Rubin’s maneuver	0 (0)	0 (0)	1
Wood’s screw maneuver	14 (12.6)	2 (1.2)	** < 0.001**
Shoulder dystocia time ≥ 90 s	13/48 (27.1)	4/46 (8.7)	**0.02**
Induction of labor	37 (33.3)	51 (31.1)	0.69
Episiotomy	44 (39.6)	48 (29.1)	0.07
Epidural analgesia	83 (74.8)	107 (64.8)	0.09
Neonatal weight (g)	3856.1 ± 396.9	3804.6 ± 390.3	0.28
Macrosomia (birthweight ≥ 90th percentile for gestational age/absolute weight ≥ 4000 g)	70 (63.1)	80 (48.7)	**0.02**
Night shift	86 (77.5)	132 (82.1)	0.54

All data are shown as number (%), mean ± standard deviation or median (range), as appropriate. Values in bold are statistically significant (*p* < 0.05)

Description of the individual complications that formed the unfavorale outcome group is presented in Table 3.

**Table 3:** Descriptionof the individual complications that formed the unfavorale outcome group

Unfavorable outcome *n* = 111	
Maternal complications	
Perineal tear grade 3	4 (3.6)
Perineal tear grade 4	1 (0.9)
Postpartum fever	11 (9.9)
Postpartum hemorrhage	25 (22.5)
Blood transfusion	15 (13.5)
Neonatal complications	
Apgar 5 min < 7	5 (4.5)
pH < 7.1	5 (4.5)
Respiratory morbidity	58 (52.2)
Cerebral morbidity	2 (1.8)
Erb’s palsy	24 (21.6)
Clavicular fracture	13 (11.7)
Necrotizing enterocolitis	0 (0)
Sepsis	4 (3.6)
Transfusion	3 (2.7)
Neonatal death	0 (0)

All data are shown as number (%). Respiratory morbidity include respiratory distress syndrome, mechanical ventilation or need for respiratory support; cerebral morbidity include intra-ventricular hemorrhage, seizures or hypoxic–ischemic encephalopathy

Table 4 presents the results of a multivariable regression analysis to identify independent associations with compositeunfavorable maternal/neonatal outcomes in deliveries complicated with shoulder dystocia. In this analysis, short maternal stature (aOR = 1.12, 95% CI 1.04–2.01), pre-gestational diabetes (aOR = 1.22, 95% CI 1.03–3.09), vacuum extraction (aOR = 1.95, 95% CI 1.22–4.36), Wood’s screw maneuver (aOR = 2.66, 95% CI 1.77–6.39), and macrosomia (aOR = 1.04, 95% CI 1.01–2.77) were associated with unfavorable maternal/neonatal outcome in deliveries complicated with shoulder dystocia.

**Table 4:** Results of a multivariable regression analysis to identify independent associations with composite unfavorable maternal/ neonatal utcomes in deliveries complicated by shoulder dystocia

Variable	aOR	95% CI
Short maternal stature (≤ 160 cm)	1.12	1.04–2.01
BMI (kg/m^2^)	NS	NS
Pre-gestational diabetes	1.22	1.03–3.09
Smoking	NS	NS
Vacuum extraction	1.95 2.66	1.22–4.36
Wood’s screw maneuver		1.77–6.39
Shoulder dystocia time ≥ 90 s	NS	NS
Macrosomia (birthweight ≥ 90th percentile for gestational age/absolute weight ≥ 4000 g)	1.04	1.01.2.77

*BMI* body mass index. Values in bold were significant in the regression analysis. NS = not significant

## Discussion

### Principal findings

This study sought to identify discerning factors contributing to adverse maternal/neonatal outcomes in instances of shoulder dystocia. Within the purview of our investigation, we identified specific predictors, namely: maternal shorter stature, pre-gestational diabetes, employment of vacuum extraction, implementation of the Wood’s screw maneuver, and neonatal macrosomia.

### Results in the context of what is known

Shoulder dystocia is an obstetrical emergency associated with adverse maternal and neonatal outcomes, and while there are instances with identifiable predictors, a substantial portion of cases remains elusive in terms of predictive factors. Numerous studies have tried to find predictors for shoulder dystocia; however, a significant proportion—ranging from 50 to 75%—of shoulder dystocia cases arises without the presence of discernible risk factors [27, 28].

Studies focusing on predictors for adverse outcomes in shoulder dystocia cases are limited. Narendran et al. [20] conducted a study in 2021 encompassing 1134 instances of shoulder dystocia, shedding light on predictive markers for neonatal brachial plexus palsy. Their findings underscored associations with diabetes, birthweight exceeding 4000 g, the act of seeking assistance during shoulder dystocia, and a shoulder dystocia duration of 120 s or more. Harari et al. [29] found in a study from 2021 that while fetal macrosomia, maternal diabetes mellitus, male gender, and advanced maternal age are associated with shoulder dystocia, no association was found between shoulder dystocia and long-term neonatal neurological morbidity. Other studies [21, 30, 31] reinforced these findings by revealing significant associations. Factors such as macrosomia, lower maternal height, elevated maternal weight, excessive maternal weight gain, as well as gestational age and parity, were implicated in neonatal birth injuries. In addition, Hehir et al. conducted a study in 2018 involving 685 cases of shoulder dystocia, identifying predictors for maternal sphincter injury. Nulliparity, operative vaginal delivery, and the utilization of internal maneuvers emerged as substantial predictors [32].

### Clinical and research implications

In the present study, we tried to establish a comprehensive framework encompassing maternal, obstetrical, and neonatal parameters collectively, as opposed to analyzing them individually. This holistic approach aims to uncover potential interconnections among these parameters, recognizing their mutual influence, and to ascertain whether they collectively correlate with unfavorable maternal and neonatal outcomes. For instance, maternal height below 160 cm is suggested as a potential risk factor for an inadequate pelvis, leading to cephalopelvic disproportion [33] and consequent shoulder dystocia. Pre-gestational diabetes is another recognized risk factor for shoulder dystocia, primarily attributed to typical fetal muscle and fat distribution patterns [34, 35] often resulting in increased birthweight [36]. Furthermore, macrosomia [37] and interventions like vacuum extraction [13] and Wood’s screw maneuver [38, 39] might contribute to traumatic labor for both the mother and the neonate. These suppositions align with the findings of our study.

## Strengths and limitations

The current study is unique in several aspects. First, it investigated a relatively large cohort of labors complicated with shoulder dystocia. Second, this study, in contrast to most previous studies, examined a large spectrum of neonatal complications. Third, this study concentrated not only on neonatal complications resulting from shoulder dystocia, but also maternal complications. Our study is not without limitations. First, we have only collected short-term neonatal outcomes. Second, since shoulder dystocia is a relatively uncommon event, and the separate adverse maternal and neonatal outcomes are each uncommon themselves, we had to apply the current methodology combining the two to a composite adverse outcome. When trying to investigate each separately, each of the important findings did not reach statistical significance. While we are conscious that using a composite outcome may be seen as a research constraint, we think it was necessary to utilize one because the individual components of the composite are uncommon problems. We have described and validated the same composite neonatal outcomes in our previous publications with other pregnancy complications [40]. Third, shoulder dystocia is a clinical diagnosis biased by the individual caregiver, and all cases participated in this study were retrieved based on this subjective computerized diagnosis. Lastly, cases of shoulder dystocia that were immediately and easily managed were probably underreported and, therefore, underrepresented in this study.

## Conclusion

In conclusion, shoulder dystocia is an unpredictable obstetrical emergency with numerous potential maternal and neonatal adverse outcomes. This study puts the focus on risk factors which are associated with unfavorable maternal and neonatal outcome in cases of shoulder dystocia: short maternal stature, pre-gestational diabetes, vacuum extraction, Wood’s screw maneuver, and macrosomia. Understanding these risk factors might help clinicians give a better consultation for women at risk of labor complicated with shoulder dystocia and avoid poor maternal and neonatal outcomes.

## Data Availability

The data that support the findings of this study are available from the corresponding author, DT, upon reasonable request.
